# Large Vaginal Hematoma in a Puerperium Patient: Treating a Delayed Diagnosis & Management Caused by an Incomplete Clinical Examination

**DOI:** 10.7759/cureus.33386

**Published:** 2023-01-05

**Authors:** Sudwita Sinha, Mukta Agarwal, Shruti Singh

**Affiliations:** 1 Obstetrics and Gynaecology, All India Institute of Medical Sciences, Patna, IND

**Keywords:** increased morbidity, puerperium, delayed diagnosis, incomplete clinical examination, pelvic hematoma

## Abstract

Here, we report a case where the lack of pelvic examination in a puerperium patient led to a delay in diagnosis and appropriate management of a large posterior vaginal wall hematoma for about a month. The patient had a history of difficult vaginal breech delivery of a macrosomic asphyxiated baby 28 days prior, following which, she started having gradually increasing distension of the abdomen, inability to void urine and pass stools by herself, and a history of fever on and off. Her family members took her to a private hospital for a consultation, where she was examined and assessed. However, a pelvic examination was not done. A CT scan of the abdomen and pelvis showed a large organized hematoma of size 13.6cm×11.1cm×10.5cm with a volume of 802 ml in the pouch of Douglas. Following this report, diagnostic laparoscopy was done on day 10 of puerperium where a large hematoma was seen beneath the peritoneum in the pouch of Douglas without any intraperitoneal collection. As the hematoma was not seen to be expanding, conservative management was done with 5 units of blood transfusion and antibiotic coverage, and the patient was discharged. However, the patient’s symptoms were not relieved due to which she presented to us on day 28 of puerperium with the same symptoms. On pelvic examination, purulent, foul-smelling discharge was present in the vagina, and a huge tense bluish bulge was seen in the posterior vaginal wall more towards the right side obliterating the whole vagina. After taking informed consent and with proper pre-operative preparations of laparotomy, the hematoma was drained vaginally, and approximately 1300 ml of blood and clots were removed, following which, the patient had a speedy recovery and relief of her symptoms.

## Introduction

The key to a clinical diagnosis is always patient history and complete clinical examination. In our present scenario where our diagnosis is inevitably guided by the availability and assistance of an armamentarium of radiological imaging techniques, we are blinded by their reporting and tend to omit important aspects of clinical examination. Although investigations are no doubt very helpful in diagnosis and management, these should be taken as complementary to our clinical assessment and should not be leading the diagnosis and management. Clinical correlation is always important. Vulvo-vaginal hematomas are not commonly encountered in obstetrical practice but if not managed correctly, can be life-threatening with serious maternal morbidity and cause maternal near-miss cases [1]. They can be managed by different treatment methods which include watchful observation, surgical drainage, ligation or angiographic embolization of bleeding vessels, packing, hysterectomy, and internal iliac artery ligation [1-3]. The correct management in a particular case depends on the experienced judgment and decision of the treating clinician based on the patient’s hemodynamic stability, the size of the hematoma, its expansion, and the presenting symptoms [3].

## Case presentation

We report a case of a parity three female who presented to the Gynaecology outpatient department on day 28 of puerperium with complaints of abdominal distension, fever, inability to pass stool, and inability to void urine following delivery. The patient had a history of difficult vaginal breech delivery (baby weight 4.2kg) of an alive, asphyxiated macrosomic baby (which had to be admitted to the neonatal intensive care unit for seven days) 28 days prior with a history of prolonged second-stage labor that approximately lasted about three hours at a primary health care center. She did not give any history of postpartum hemorrhage but she had complaints of increasing weakness following delivery. After delivery, the patient was unable to pass urine by herself following which she was catheterized and sent home. After a few days, she started having distension of the abdomen, inability to pass stool, and inability to void urine by herself on the removal of the catheter. She also started having a fever on and off. She was taken to a private hospital for a checkup where she was examined and assessed. She was conscious and oriented. She was admitted to the ICU, and blood investigations and radiological imaging were done. However, a pelvic examination was not conducted. She was started on intravenous antibiotics and fluids and received 5 units of blood transfusion due to severe anemia. Ultrasonography of the abdomen and pelvis showed pelvic hematoma. Contrast-enhanced CT scan of the abdomen and pelvis was done for confirmation of USG finding of hematoma, and to measure its extent.

Based on these investigation reports, the patient was told that she needed surgery for further evaluation. Diagnostic laparoscopy was done on day 10 of puerperium which showed a normal uterus and ovaries. The right fallopian tube was mildly dilated. There was no collection in the broad ligament. A large hematoma was seen beneath the rectouterine pouch more towards the right side without any intraperitoneal collection. There was no active bleeding or oozing and the hematoma was not expanding. So no further interventions were made.

The patient was managed conservatively with intravenous antibiotics, fluids, and blood transfusion and was discharged after a week of hospitalization. However, the patient did not have relief from her symptoms. So she came to our outpatient department on day 28 of puerperium with complaints of inability to pass stool for 20 days, inability to void urine for two days, and a history of fever on and off. On examination, she was conscious and oriented. There was mild pallor. Vitals were stable. Systemic examination revealed no abnormality. The abdomen was soft and non-tender with no organomegaly and bowel sounds were auscultated. On pelvic examination, external genitalia was normal looking. On per speculum examination, a foul-smelling, copious, purulent discharge was seen in the vagina and a huge tense bulge of around 10cm ×12cm was seen in the posterior vaginal wall which was more towards the right side with upper margins not reachable. The mass was completely obliterating the vagina due to which the cervix could not be seen. On bimanual examination, the uterus could not be assessed properly. A provisional diagnosis of right postero-lateral vaginal wall hematoma was made.

The patient's blood group was determined to be A positive, viral markers were negative, and hemoglobin was 10.8 g/dl (after 5 units of blood transfusion). Contrast-enhanced CT scan showed a bulky postpartum uterus with a collection measuring 7.5cm×4cm in the endometrial cavity suggestive of a blood clot. It also showed a large well defined loculated collection of size 13.6cm×11.1cm×10.5cm, a volume of 802 ml, and with plenty of septations and internal echoes involving the rectouterine pouch causing moderate indentation over the urinary bladder, rectum, sigmoid colon, and right distal ureter suggestive of an organized hematoma. The patient was planned and prepared for hematoma drainage.

The patient was given intravenous antibiotic coverage and hematoma drainage was planned after proper preparation. With full asepsis and taking all antiseptic precautions, the incision was made and the hematoma was drained off approximately 1300 ml of blood and clots. Loculi were broken. Debridement was done with normal saline. Seropurulent discharge coming out of the uterine cavity was suctioned out and sent for culture sensitivity and was confirmed to be sterile. Haemostasis was achieved with sutures. A corrugated drain was kept in situ. Dead space was obliterated. The vagina was packed with roller gauze to assist in further hemostasis and the pack was removed after 24 hours. Close monitoring of the operated site, vital signs, and urinary output was done to rule out any further bleeding. Broad-spectrum empirical antibiotics and analgesics were administered. Post-drainage, regular vaginal douching was done with betadine solution. Sitz bath was done twice daily. The corrugated drain was removed after 48 hours. Psychiatry consultation was prescribed for her depression which the patient developed due to the prolonged stay at the hospital.

The patient had a speedy recovery following hematoma drainage with total relief of her symptoms. She was able to self-void after the removal of the urethral catheter 24 hours after drainage. She was able to pass stool on the second day following drainage. She was discharged on the fifth day following drainage in stable condition. On the follow-up seven days after discharge, all of the patient's symptoms had subsided and she was able to return to normal daily activities.

## Discussion

Postpartum vulvovaginal hematomas are an uncommon entity in obstetrical practice and if not recognized early and managed appropriately can lead to life-threatening situations, serious maternal morbidity, and maternal near-miss cases. Incidence is reported to be 1:500 to 1:12500 vaginal births while clinically significant hematoma requiring surgical drainage is seen in 1:1000 vaginal birth cases [1]. Delays in diagnosis and management can be due to non-specific symptoms and concealed nature of bleeding [1]. Vaginal hematomas from concealed bleeding can reach massive size before expansion stops as the subcutaneous tissue in the vagina is quite pliable [1]. There is a large potential space adjacent to the vaginal wall mucosa extending from the vulva inferiorly to the extraperitoneal space superiorly bound by peritoneal reflections on the bladder, uterus, rectum, and broad ligament [2].

Causes of vulvovaginal hematomas include spontaneous or iatrogenic injury to blood vessels (branches of the internal pudendal artery), pseudoaneurysm, or traumatic arteriovenous fistula [3]. Risk factors include nulliparity, precipitate labor, macrosomic babies, prolonged second-stage labor, instrumental vaginal deliveries like forceps delivery or ventouse delivery, poorly repaired lacerations or episiotomies, hypertensive disorders of pregnancies, coagulopathy or vulvar varicosities [1,3,4]. In our case, a history of prolonged second-stage labor and a macrosomic baby was present. Spontaneous hematomas are more commonly right-sided as vulvar varicosities are more commonly on the right side due to dextro-rotation of the uterus.

The key to diagnosis is the patient’s symptoms (pain, vulvovaginal swelling, urologic symptoms like inability to pass urine and rectal tenesmus, occlusion of vaginal orifice causing retention of urine, hypovolemic shock, and collapse) and pelvic examination [1]. The importance of complete clinical examination is well-known. Half the diagnosis is reached by history and clinical examination alone. In the above case, the patient had to undergo costly radiological investigations like a contrast-enhanced CT scan of the abdomen and pelvis to diagnose the hematoma, which could have been easily diagnosed had the pelvic examination been done. Furthermore, if an examination had been done, proper management would have been initiated earlier for her symptoms, and diagnostic laparoscopy, which added further financial burden to the patient, would not have been necessary. In this era of clinical practice, where investigations and imaging techniques are readily available and are inevitably used to confirm a diagnosis, decide management, and follow-up, it has led to the subconscious omitting of certain aspects of clinical examination. We are becoming too dependent on investigations. Although they are valuable, clinical examinations should not be forgotten.

Management of vulvovaginal hematomas depends on the size of the hematoma, the hemodynamic stability of the patient, and available medical facilities [3]. Conservative management can be done for small non-expanding and asymptomatic hematomas including sitz baths, ice packs, antibiotics, analgesics, and blood transfusion if required [3]. Large or expanding hematomas are managed surgically either by exploration or by arterial embolization [3]. Surgical exploration consists of incision and drainage, ligating bleeding vessels, obliterating all dead spaces, achieving hemostasis, and placing a drainage tube or vaginal pack [3]. This is what was done for our patient. Surgical exploration also prevents the risk of necrotizing fasciitis due to pressure necrosis of surrounding tissues [3]. Selective arterial embolization of bleeding vessels is required in other cases whereas a few exceptional cases might need a hysterectomy or internal iliac artery ligation.

Awoleke et al. presented a case of vulvovaginal infra-levator hematoma mimicking the second stage of labor with persistent painful bearing down efforts even after successful delivery of the baby, which was managed surgically [1]. Nnaji et al. reported a case of vulval hematoma of 12cm×10cm following spontaneous vaginal delivery that was managed conservatively [4]. Tseng et al. reported three cases of vaginal hematoma; first, of an increasing hematoma of 12cm size on day two of puerperium, second with an impending rupture, and a third postpartum bilateral hematoma; all were managed by debridement and arterial embolization [5]. Stobie et al.reported a case of large paravaginal hematoma measuring 12cm×8cm×7cm causing postpartum hemorrhage which was managed conservatively [2]. Tilahun et al. reported a case of spontaneous vulvar hematoma of size 20cm×12cm as a cause of a maternal near-miss that was surgically drained [3]. Ndu-Akinla et al. reported a case of vulvovaginal hematoma in a puerperium patient of size 12cmx14 cm which was managed by evacuation [6]. Kehila et al. reported two cases of postpartum vaginal hematomas managed by arterial embolization [7]. Dahdouh et al. reported a case of postpartum vulvovaginal hematoma which was managed by angiographic embolization [8].

## Conclusions

To conclude, optimal and timely diagnosis and management of postpartum vulvar and vaginal hematomas are necessary to avoid maternal morbidity. Patient’s symptoms and clinical examination are key to early diagnosis. Proper attention to the patient’s symptoms and clinical examination including pelvic examination is a must for early diagnosis and management. Management can be either conservative, surgical drainage, or embolization depending on the case.

Although advanced imaging techniques have developed, these cannot replace a complete clinical examination. No part of the clinical examination should be deemed unnecessary and omitted. The importance of a pelvic examination should not be dismissed. Provisional diagnosis should be reached based on history and clinical examination alone and investigations should only be used to confirm or refute the diagnosis and not as a means to reach a diagnosis. Proper attention should be given to the patient’s symptoms while managing a patient. Physicians should treat the patient, and not their investigations.
